# Long-term warming in a Mediterranean-type grassland affects soil bacterial functional potential but not bacterial taxonomic composition

**DOI:** 10.1038/s41522-021-00187-7

**Published:** 2021-02-08

**Authors:** Ying Gao, Junjun Ding, Mengting Yuan, Nona Chiariello, Kathryn Docherty, Chris Field, Qun Gao, Baohua Gu, Jessica Gutknecht, Bruce A. Hungate, Xavier Le Roux, Audrey Niboyet, Qi Qi, Zhou Shi, Jizhong Zhou, Yunfeng Yang

**Affiliations:** 1 Institute of Desertification Studies, Chinese Academy of Forestry, Beijing, China; 2 State Key Joint Laboratory of Environment Simulation and Pollution Control, School of Environment, Tsinghua University, Beijing, China; 3 Key Laboratory of Dryland Agriculture, Ministry of Agriculture of the People’s Republic of China, Institute of Environment and Sustainable Development in Agriculture, Chinese Academy of Agricultural Sciences, Beijing, China; 4 Institute for Environmental Genomics and Department of Microbiology and Plant Biology, University of Oklahoma, Norman, OK USA; 5 Department of Global Ecology, Carnegie Institution for Science, Stanford, CA USA; 6 Department of Biological Sciences, Western Michigan University, Kalamazoo, MI USA; 7 Environmental Sciences Division, Oak Ridge National Laboratory, Oak Ridge, TN USA; 8 Department of Soil Ecology, Helmholtz Centre for Environmental Research - UFZ, Halle, Germany; 9 Department of Soil, Water, and Climate, University of Minnesota, Twin Cities, Saint Paul, MN USA; 10 Center for Ecosystem Science and Society, Northern Arizona University, Flagstaff, AZ USA; 11 Department of Biological Sciences, Northern Arizona University, Flagstaff, AZ USA; 12 Mirobial Ecology Centre LEM, INRA, CNRS, University of Lyon, University Lyon 1, UMR INRA 1418, Villeurbanne, France; 13 Institut d’Ecologie et des Sciences de l’Environnement de Paris (Sorbonne Université, CNRS, INRA, IRD, Université Paris Diderot, UPEC), Paris, France; 14 AgroParisTech, Paris, France; 15 Earth Sciences Division, Lawrence Berkeley National Laboratory, Berkeley, CA USA

**Keywords:** Microbiome, Soil microbiology

## Abstract

Climate warming is known to impact ecosystem composition and functioning. However, it remains largely unclear how soil microbial communities respond to long-term, moderate warming. In this study, we used Illumina sequencing and microarrays (GeoChip 5.0) to analyze taxonomic and functional gene compositions of the soil microbial community after 14 years of warming (at 0.8–1.0 °C for 10 years and then 1.5–2.0 °C for 4 years) in a Californian grassland. Long-term warming had no detectable effect on the taxonomic composition of soil bacterial community, nor on any plant or abiotic soil variables. In contrast, functional gene compositions differed between warming and control for bacterial, archaeal, and fungal communities. Functional genes associated with labile carbon (C) degradation increased in relative abundance in the warming treatment, whereas those associated with recalcitrant C degradation decreased. A number of functional genes associated with nitrogen (N) cycling (e.g., denitrifying genes encoding nitrate-, nitrite-, and nitrous oxidereductases) decreased, whereas *nifH* gene encoding nitrogenase increased in the warming treatment. These results suggest that microbial functional potentials are more sensitive to long-term moderate warming than the taxonomic composition of microbial community.

## Introduction

Accumulating concentrations of greenhouse gases in the atmosphere are increasing global surface temperature, which is expected to persist in the coming decades^1^. Warming often stimulates plant photosynthesis, biomass, and growth^2–5^, and alters plant community composition^6,7^. Soil C and N cycling are also altered by warming^8–11^. In comparison, there is less information on responses of the soil taxonomic and functional genes to warming. More precisely, many studies have analyzed the effect of brief heat shocks (often applied over a few minutes or hours)^12,13^; or warm climate spells (typically applied over a few days or weeks)^14,15^; on soil microbial communities. But studies analyzing the effect of moderate and progressive warming on a long term (over years to decades), as currently experienced due to global warming, are very scarce.

Soil microbial communities are vastly diverse, and are key drivers of soil biogeochemical cycling^16,17^. Warming can increase soil microbial activities, change microbial biomass, and community composition^15,18–20^, but the magnitude of warming effects vary by climate regions and ecosystem type. For example, a meta-analysis of 64 studies showed that warming increased microbial biomass in grasslands (mean of 8.3%)^21^. As microbial processes play a substantial role in ecosystem models^22,23^, advancing our knowledge of soil microbial responses to moderate, long-term warming is crucial for predicting ecological consequences of future climate changes.

Regions with a Mediterranean climate, characterized by hot, dry summers and cool, wet winters, are particularly vulnerable to global warming^24–26^, owing to warming-induced changes such as water scarcity. The Jasper Ridge Global Change Experiment (JRGCE) investigates the impacts of multiple global environmental changes^27,28^, including warming, on a Californian annual grassland experiencing a Mediterranean climate. In this ecosystem, relatively short-term warming (i.e., 2–6 years) had accelerated the flowering and greening of the canopy plants, increased forb production and abundance, stimulated arbuscular mycorrhizal fungi (AMF) hyphal length and total ammonia-oxidizing bacteria (AOB) abundance, and decreased the relative abundance of methane-oxidizing bacteria^27–35^. However, it had no impacts on plant biomass, shoot, root, forbs or grass biomass, N cycling process rates, and enzyme activities. At longer time scale (8–9 years), microbial community composition assessed by PLFAs fluctuated greatly over multiple years^36^. Although microbial communities were mostly not responsive to the warming, community composition was changed by warming in 2006. Soil total C concentration also remained unaltered, but the fraction of C derived from microbes, i.e., residues quantified by the microbial amino sugars, significantly decreased in the soils exposed to warming^37^. Those findings are consistent with accumulating evidence that warming often yields different results over the short- and long-terms. For instance, in a temperate deciduous forest, warming had no effect on the composition of the soil bacterial community after 5 or 8 years, but changes were apparent after 20 years^38^. Another 26-year warming study conducted in a mid-latitude hardwood forest also showed that soil microbial responses to warming were different over time^39^. Thus, it is essential to gain further insights into long-term ecosystem responses to warming^40^.

Accumulative evidence has noted a decoupling of microbial taxonomy and functional structure^41,42^. Therefore, it is necessary to examine both microbial taxonomy and functional genes. Here, we present data examining the functional and taxonomic composition of soil microbial communities sampled after 14 years of warming at the JRGCE (0.8–1.0 °C for 10 years and then 1.5–2.0 °C for 4 years). We used Illumina sequencing of 16S rRNA gene amplicons to examine the taxonomic composition of soil bacterial community. We used GeoChip 5.0 to examine functional gene composition derived from bacteria, fungi, and archaea. We also measured plant and soil geochemical variables to examine to what extent changes in taxonomic and functional genes of the soil microbial community in response to warming might be related to changes in plant/soil variables. We aim to test the following hypotheses: (1) Although the warming treatment is moderate, 14 years of warming may be sufficient to induce significant changes in plant communities, microbial communities, and soil geochemical variables; and (2) Microbial responses to warming are different at the taxonomic and functional gene levels.

## Results

### Plant and soil variables

Warming significantly increased the average soil temperature during the year and month of sampling by 1.1 °C at 10-cm depth (Table 1). Perennial forbs biomass (PFB) decreased 49.8% whereas both annual forbs biomass (AFB) and litter biomass (LB) increased 17.1% and 19.9%, respectively, due to warming, though these changes represent a non-significant trend (*P* > 0.05). Annual grass biomass (AGB), total aboveground biomass (TAGB), total shallow root biomass (TSRB), and fine root biomass (FRB) remained unchanged. Plant standing biomass (PSB), a measure of plant growth consisting of the sum of aboveground biomass (grass and forbs), litter, shallow roots, and fine roots, was not altered by the warming treatment. Soil variables, such as soil moisture, pH, total N, total C, and C:N ratio did not differ between warmed and control plots. Extractable soil NO_3_-N increased 2-fold while extractable soil NH_4_-N decreased 16.7% in response to warming; however, these changes were not significant (*P* > 0.05).

**Table 1:** The mean values and coefficient of variation (CV) of environmental variables in warmed and control samples (*n* = 4).

Environmental variables	Control	CV	Warming	CV	Percentage change	*P*-value
Annual mean soil temperature (10 cm, °C)	18.33	1.83%	19.42	2.68%	5.95%	**0.013**^**a**^
April mean soil temperature (10 cm, °C)	15.34	2.69%	16.46	3.45%	7.30%	**0.019**
AGB (g m^−2^)	153.23	37.66%	161.17	86.11%	5.18%	0.920
AFB (g m^−2^)	106.95	67.23%	125.19	54.02%	17.05%	0.724
PFB (g m^−2^)	22.16	119.08%	11.12	129.50%	−49.82%	0.490
LB (g m^−2^)	117.46	32.64%	140.85	28.88%	19.91%	0.435
TAGB (g m^−2^)	283.17	34.55%	299.06	34.64%	5.61%	0.831
TSRB (g m^−2^)	11.08	90.82%	11.81	126.09%	6.59%	0.938
FRB (g m^−2^)	4.70	23.97%	4.98	36.76%	5.96%	0.801
PSB (g m^−2^)	416.40	18.69%	456.70	22.81%	9.68%	0.558
TOC (mg kg^−1^ dry soil)	24.45	16.80%	27.52	14.25%	12.56%	0.321
Soil pH	6.03	2.33%	6.23	1.78%	3.32%	0.064
Moisture (%)	7.98	38.78%	7.83	25.13%	−1.88%	0.935
NO_3_^−^ (mg L^−1^)	79.68	32.93%	240.69	151.02%	202.07%	0.411
NH_4_^+^ (mg L^−1^)	401.74	44.08%	334.80	83.42%	−16.66%	0.700
TC (g kg^−1^ dry soil)	12.62	12.32%	12.73	17.34%	0.87%	0.942
TN (g kg^−1^ dry soil)	1.21	8.29%	1.19	13.15%	−1.65%	0.876
CNR	10.43	7.52%	10.61	5.94%	1.73%	0.727

*AGB* annual grass biomass, *AFB* annual forbs biomass, *PFB* perennial forbs biomass, *LB* litter biomass, *TAGB* total aboveground biomass, *TSRB* total shallow root biomass, *FRB* fine root biomass, *PSB* plant standing biomass, *TOC* total organic carbon, *TC* total carbon, *TN* total nitrogen, *CNR* carbon to nitrogen ratio.

^a^Significant values, determined by two-tailed paired *t*-tests, are indicated in bold font.

### Taxonomic composition of soil microbial communities

Among OTUs obtained from each sample, 7.7% were unclassifiable (not represented in reference sequence databases). Microbial taxonomic composition and alpha-diversity did not differ between the warming treatment plots and the control plots (Supplementary Fig. 1a and Supplementary Table 1), which was supported by results of three nonparametric multivariate statistical tests (*P* > 0.05, Supplementary Table 2).

The classified OTUs were divided into 2 archaeal phyla and 21 bacterial phyla. The most abundant bacterial phyla were *Proteobacteria* (27.4%), *Acidobacteria* (18.7%), *Actinobacteria* (17.9%), and *Verrucomicrobia* (7.7%, Supplementary Fig. 2). There were no significant changes in the relative abundances of any phyla or finer taxonomic levels of genus, family, order, or class by warming.

### Microbial functional genes

A total of 61,908 functional genes, classified into 54,554 bacterial genes, 4532 fungal genes, 1868 archaeal genes, 400 viral genes, 209 other eukaryotic genes, and 345 unclassified genes, were detected by GeoChip 5.0. Non-metric multidimensional scaling (NMDS) showed that microbial functional gene composition was affected by warming (Supplementary Fig. 1b). The results of three nonparametric multivariate statistical tests (Adonis, ANOSIM, and MRPP) revealed significant shifts in the functional gene composition of microbial communities in response to warming (*P* < 0.05, Supplementary Table 2). In contrast, microbial functional diversity and evenness were unchanged (Supplementary Table 1). Functional gene communities of bacteria, archaea, and fungi were changed by warming (Supplementary Fig. 3), which were also verified by three nonparametric multivariate statistical tests (*P* < 0.05, Supplementary Table 2).

#### C cycling

We used the same nonparametric multivariate tests (Adonis, ANOSIM, and MRPP) to determine that the centroids for 19 of 63 functional genes detected by probes related to C cycling were different (unadjusted *P* < 0.05, Table 2) between ‘warmed’ and ‘control’ plots, indicating a significant shift in the composition of C cycling genes in response to warming. These genes include *pulA* encoding pullulanase, *amyX* encoding amylopullulanase, *nplT* encoding neopullulanase, *ara* encoding arabinofuranosidase, *xylA* encoding xylosidase, exoglucanase gene, ligninase gene, and endochitinase gene, which were significantly altered by the warming treatment (Table 2). However, these differences were not significant after correcting error rates for multiple comparisons (adjusted *P* > 0.05, Table 2).

**Table 2:** Significance tests of the warming effects on the C degradation and N cycling gene compositions with three nonparametric statistical analyses (*n* = 4).

	Categories	Gene name	Adonis^a^			ANOSIM^b^			MRPP^c^		
			*R*^2^	*P*^d^	Adjusted *P*-value^e^	*R*	*P*	Adjusted *P*-value	δ	*P*	Adjusted *P*-value
C degradation	Sucrose	invertase	0.102	0.658	0.703	−0.083	0.730	0.767	0.145	0.692	0.715
		dextranase	0.021	0.855	0.869	0.125	0.202	0.283	0.025	0.264	0.347
	Lactose	lactase	0.381	0.087	0.161	0.354	0.064	0.144	0.120	0.063	0.143
	Glucose	Glucose	0.176	0.315	0.361	0.188	0.144	0.239	0.118	0.276	0.348
	Agar	beta_agarase	0.357	**0.029**	0.081	0.385	**0.036**	0.109	0.116	**0.026**	0.105
	Starch	*pulA*	0.306	**0.029**	0.081	0.281	**0.029**	0.109	0.110	**0.035**	0.105
		*amyA*	0.245	0.091	0.161	0.188	0.123	0.215	0.124	0.096	0.178
		glucoamylase	0.221	0.146	0.204	0.104	0.268	0.345	0.106	0.173	0.242
		*amyX*	0.550	**0.027**	0.081	0.552	**0.032**	0.109	0.160	**0.024**	0.105
		*cda*	0.293	**0.030**	0.081	0.229	**0.022**	0.109	0.135	**0.021**	0.105
		isopullulanase	0.409	**0.031**	0.081	0.375	**0.036**	0.109	0.093	**0.039**	0.105
		*nplT*	0.336	**0.028**	0.081	0.406	**0.019**	0.109	0.132	**0.040**	0.105
		*apu*	0.236	0.115	0.186	−0.021	0.617	0.682	0.055	0.369	0.423
	Hemicellulose	*ara*	0.277	**0.027**	0.081	0.313	**0.026**	0.109	0.105	**0.035**	0.105
		xylanase	0.250	0.089	0.161	0.208	0.159	0.25	0.126	0.154	0.237
		*xylA*	0.295	**0.030**	0.081	0.302	**0.035**	0.109	0.127	**0.024**	0.105
		mannanase	0.243	0.142	0.204	0.052	0.296	0.366	0.109	0.161	0.242
		xylose_reductase	0.182	0.113	0.186	−0.031	0.700	0.747	0.363	0.346	0.411
	Cellulose	endoglucanase	0.271	**0.030**	0.081	0.281	0.056	0.136	0.110	0.065	0.143
		cellobiase	0.315	**0.027**	0.081	0.313	**0.034**	0.109	0.094	**0.032**	0.105
		exoglucanase	0.300	**0.030**	0.081	0.323	**0.042**	0.115	0.120	**0.029**	0.105
	Pectin	pec_CDeg	0.253	0.169	0.22	0.250	**0.038**	0.109	0.124	0.119	0.203
		pectate_lyase	0.246	0.090	0.161	0.260	0.063	0.144	0.113	0.112	0.196
		*pme*	0.287	**0.028**	0.081	0.281	0.052	0.131	0.122	**0.029**	0.105
		*rgh*	0.274	0.056	0.128	0.125	0.201	0.283	0.125	0.081	0.159
		endopolygalacturonase	0.209	0.170	0.22	0.198	0.091	0.174	0.138	0.127	0.211
		pectin_lyase	0.354	0.146	0.204	0.167	0.143	0.239	0.153	0.140	0.222
		*rgl*	0.297	**0.029**	0.081	0.240	0.089	0.174	0.115	0.023	0.105
		*RgaE*	0.306	**0.030**	0.081	0.292	0.051	0.131	0.110	**0.029**	0.105
		pectinase	0.186	0.314	0.361	−0.083	0.886	0.886	0.146	0.383	0.431
		exopolygalacturonase	0.391	**0.027**	0.081	0.438	**0.035**	0.109	0.119	**0.029**	0.105
		*Pg*	0.380	**0.027**	0.081	0.469	**0.023**	0.109	0.140	**0.030**	0.105
	Lignin	phenol	0.305	**0.030**	0.081	0.333	**0.035**	0.109	0.114	**0.035**	0.105
		*glx*	0.195	0.233	0.282	0.115	0.229	0.314	0.125	0.288	0.356
		*mnp*	0.201	0.271	0.322	0.031	0.494	0.583	0.133	0.446	0.493
		ligninase	0.589	**0.028**	0.081	0.667	**0.034**	0.109	0.141	**0.021**	0.105
	Vanillin/lignin	*vanA*	0.209	0.171	0.22	0.188	0.078	0.164	0.094	0.179	0.245
		*vdh*	0.134	0.573	0.633	0.094	0.25	0.335	0.093	0.335	0.406
	Inulin	inulinase	0.316	0.142	0.204	0.302	0.122	0.215	0.086	0.066	0.143
		exoinulinase	0.032	0.944	0.944	−0.073	0.5	0.583	0.043	0.570	0.609
	Cutin	cutinase	0.227	0.228	0.282	0.010	0.432	0.523	0.085	0.275	0.348
	Chitin	endochitinase	0.274	**0.026**	0.081	0.292	**0.033**	0.109	0.114	**0.031**	0.105
		chitinase	0.229	0.089	0.161	0.208	0.167	0.257	0.096	0.171	0.242
		acetylglucosaminidase	0.248	0.064	0.139	0.271	0.074	0.161	0.095	0.068	0.143
		exochitinase	0.259	0.088	0.161	0.323	0.024	0.109	0.100	0.043	0.108
		chitin_deacetylase	0.23	0.143	0.204	0.198	0.175	0.263	0.141	0.166	0.242
	Glyoxylate cycle	*AceA*	0.278	**0.027**	0.081	0.260	**0.024**	0.109	0.111	**0.023**	0.105
		*AceB*	0.281	0.057	0.128	0.240	0.104	0.193	0.104	0.046	0.111
	Camphor	camDCAB	0.087	0.768	0.806	−0.073	0.797	0.823	0.051	0.706	0.717
	Alginate	alginase	0.254	0.053	0.128	0.167	0.285	0.359	0.129	0.091	0.174
	Hyaluronic acid	hyaluronidase	0.210	0.231	0.282	−0.052	0.657	0.714	0.132	0.364	0.423
	Heparin	Heparinase	0.408	**0.028**	0.081	0.417	**0.032**	0.109	0.110	**0.030**	0.105
	Lipids	lipase	0.019	0.48	0.54	−0.063	0.596	0.671	0.104	0.519	0.564
	Protein	metalloprotease	0.403	**0.031**	0.081	0.510	**0.037**	0.109	0.121	**0.037**	0.105
		protease	0.274	**0.029**	0.081	0.313	**0.033**	0.109	0.150	**0.032**	0.105
	Phospholipids	phospholipase_C	0.310	0.054	0.128	0.302	**0.031**	0.109	0.117	**0.027**	0.105
		phospholipase_A2	0.309	0.092	0.161	0.385	0.083	0.169	0.130	0.078	0.159
		phospholipase_D	0.271	0.112	0.186	0.177	0.152	0.246	0.130	0.108	0.194
	Terpenes	CDH	0.109	0.651	0.703	−0.042	0.539	0.617	0.081	0.653	0.686
		limEH	0.235	0.150	0.205	0.156	0.184	0.27	0.085	0.185	0.248
		LMO	0.065	0.825	0.852	−0.135	0.862	0.876	0.115	0.831	0.831
	Other	Sulfhydryl	0.527	**0.029**	0.081	0.448	**0.027**	0.109	0.140	**0.037**	0.105
		alpha_galactosidase	0.313	0.141	0.204	0.083	0.263	0.345	0.128	0.141	0.222
N cycling	Ammonification	*ureC*	0.314	**0.027**	0.07	0.281	**0.028**	0.14	0.091	**0.034**	0.111
		*gdh*	0.338	0.086	0.12	0.302	0.054	0.153	0.119	**0.033**	0.111
	Nitrogen fixation	*nifH*	0.310	**0.029**	0.07	0.333	**0.028**	0.14	0.132	**0.031**	0.111
	Nitrification	*amoA*	0.302	**0.029**	0.07	0.292	**0.026**	0.14	0.118	**0.031**	0.111
		*hao*	0.250	0.147	0.156	0.208	0.174	0.261	0.136	0.168	0.189
	Denitrification	*norB*	0.262	0.114	0.133	0.115	0.265	0.367	0.134	0.118	0.158
		*nosZ*	0.332	0.058	0.104	−0.01	0.496	0.496	0.106	0.154	0.185
		*narG*	0.267	0.057	0.104	0.25	0.089	0.178	0.107	0.066	0.119
		*nirK*	0.301	**0.028**	0.07	0.219	0.061	0.153	0.103	0.051	0.115
		*nirS*	0.324	**0.029**	0.07	0.24	0.068	0.153	0.103	**0.037**	0.111
	Anammox	*hzo*	0.256	0.118	0.133	0.052	0.316	0.379	0.113	0.121	0.158
	Dissimilatory N reduction	*napA*	0.306	**0.030**	0.07	0.01	0.475	0.496	0.134	0.313	0.331
		*nrfA*	0.300	0.084	0.12	0.052	0.301	0.379	0.113	0.123	0.158
	Assimilatory N reduction	*nir*	0.163	0.345	0.345	0.031	0.39	0.439	0.128	0.450	0.45
		*nasA*	0.335	**0.031**	0.07	0.188	0.168	0.261	0.097	0.092	0.151
		*nirB*	0.418	**0.031**	0.07	0.365	**0.031**	0.14	0.093	**0.030**	0.111
		*nirA*	0.331	0.087	0.12	0.333	0.059	0.153	0.094	**0.049**	0.115
	N Assimilation	Nitrate reductase	0.336	0.115	0.133	0.271	0.135	0.243	0.108	0.066	0.119

^a^Nonparametric multivariate analysis of variance (MANOVA) with the adonis function.

^b^Analysis of similarities.

^c^Multiple response permutation procedure, a nonparametric procedure that does not depend on assumptions such as normally distributed data or homogeneous variances, but rather depends on the internal variability of the data.

^d^Significant effects are indicated in bold font.

^e^Benjamini–Hochberg method was adopted for correcting error rates of multiple comparisons.

**Fig. 1:**
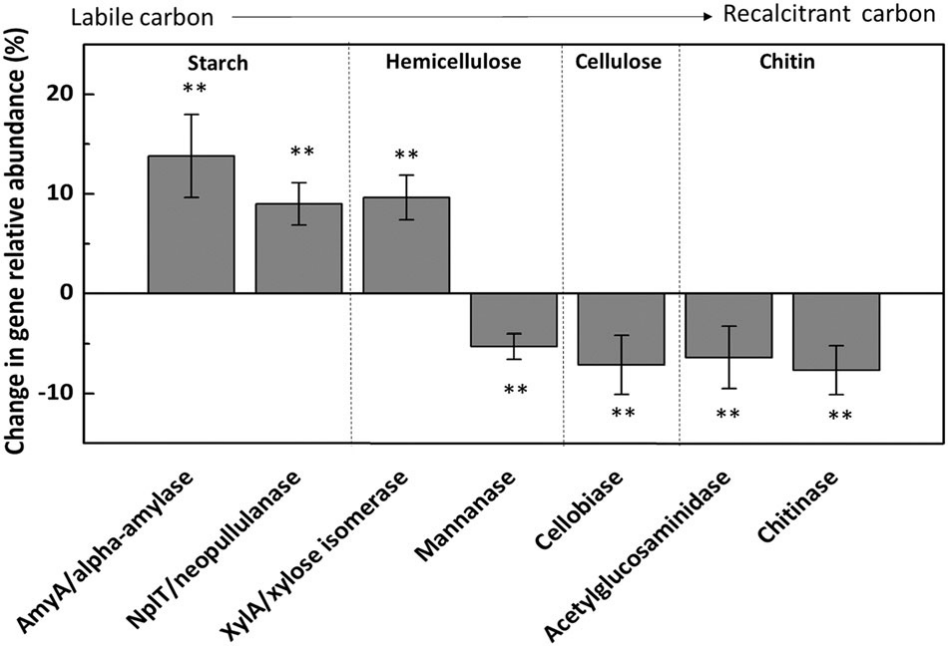
The warming effect on carbon (C) degradation genes. The complexity of C is arranged in the order from labile to recalcitrant. Mean abundances of C degradation genes are compared between warmed and control samples, which were calculated as (warming−control)/control × 100%. Error bars represent standard error (*n* = 4). The differences between warming and control were analyzed by two-tailed paired Student’s *t*-tests (*n* = 4). **P < 0.05, ***P < 0.01.

Only seven C cycling genes were significantly changed in abundance (Fig. 1), as examined by two-tailed paired Student’s *t*-test. Genes exhibiting increased abundances in the warming treatment included *amyA* encoding alpha-amylase, *nplT* encoding neopullulanase for starch degradation, and *xylA* encoding xylose isomerase for hemicellulose degradation. Genes with decreased abundance included mannanase gene for hemicellulose degradation, cellobiase gene for cellulose degradation, acetylglucosaminidase, and chitinase genes for chitin degradation.

#### N cycling

Similar to C cycling, we conducted three nonparametric multivariate statistical tests to examine changes in functional composition of genes related to N cycling. We found that 8 genes (i.e., *ureC* encoding urease, *nifH* encoding dinitrogenase reductase, *amoA* encoding ammonia monooxygenase, *nirB* encoding nitrite reductase, *napA* encoding nitrate reductase, *nasA* encoding nitrate reductase, *nirK*/*nirS* encoding nitrite reductase) were altered by warming (unadjusted *P* < 0.05, Table 2).

**Fig. 2:**
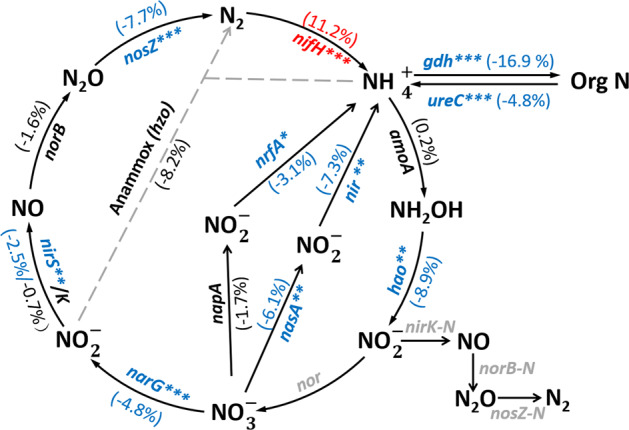
The warming effect on nitrogen (N) cycle genes. The percentages in brackets indicate changes in mean abundances of functional genes between warmed and control samples, with red and blue colors representing increase and decrease, respectively. Black color represents no significant changes in the abundances by warming. Gray color represents genes which are not targeted by the version of GeoChip used here, not detected or not applicable. The significant differences between warmed and control sites were analyzed by two-tailed paired Student’s *t*-tests (*n* = 4). *P < 0.10, **P < 0.05, ***P < 0.01.

A number of functional genes associated with N cycling decreased in relative abundance with long-term warming (Fig. 2). In particular, *gdh* encoding glutamate dehydrogenases for conversion of glutamate to alpha-ketoglutarate significantly decreased by 16.9%, and *ureC* encoding a subunit of urease significantly decreased by 4.8%. The relative abundance of denitrifying genes coding for nitrate- (*narG*, −4.8%), nitrite- (*nirS*, −2.5%) and nitrous oxidereductases (*nosZ*, −7.7%) all significantly decreased in warmed plots. Denitrifying enzyme activity (DEA) was considerably, albeit insignificantly, changed (17.7%, *P* = 0.52, Supplementary Table 3) in warmed plots. The relative abundance of the *amoA* gene harbored by archaeal and bacterial ammonia oxidizers did not change significantly whereas another nitrification gene, namely *hao* encoding hydroxylamine oxygenase, significantly decreased in warmed plots (−8.9%). Nitrifying enzyme activity (NEA) remained unchanged (7.1%, *P* > 0.05, Supplementary Table 3). Warming also induced significant decreases in *nrfA*, *nasA*, and *nir* genes associated with dissimilatory and assimilatory N reduction. In contrast, *nifH* encoding nitrogenase—a key gene for N fixation—increased by 11.2% (*P* < 0.01) in warmed plots (Fig. 2).

## Discussion

Our results showed that after 14-year of experimental treatment, warming had no detectable effect on the taxonomic composition and alpha-diversity of soil bacterial and archaeal communities (Supplementary Fig. 1a and Supplementary Table 1). Similarly, a previous study conducted at the same site found that 6 years of warming did not broadly change microbial community composition, as assessed by PLFA markers^36^. The resistance of microbial communities to warming at the taxonomic level might be attributable to several mechanisms. First, microbial communities in Mediterranean-type grassland soils experience considerable yearly and monthly fluctuations in environmental variables, such as temperature and moisture^43^, N supply^44^, and plant growth^36^, which can lead to substantial variations in soil microbial communities^36^. This might explain their weak taxonomic response to warming, as soil microorganisms adapted to frequent environmental changes may better resist further environmental stresses or changes^14,45^. However, treatment effects may play an important role in shaping the microbial communities and soil variables in the long-term^46^, since our data represent the aggregate effects of 14-year exposure to continuous warming manipulation despite a single time point sampling. Second, our warming treatment might be not sufficient in magnitude to evoke a significant response in microbial community composition and alpha-diversity because the increase in soil temperature was only 1–2 °C, smaller than the magnitude of intra-annual fluctuations in temperature at the time of peak plant biomass^36^. Although warming by infrared heaters typically decreases soil moisture, our study site is quite dry, with average soil moisture in April of 7.9%. Therefore, mild increase in soil temperature of 1–2 °C is insufficient to induce notable changes in soil moisture in April (Table 1)^47^, and changes in microbial taxonomic compositions^48,49^. Third, the soil at our study site was warmed 1.0 °C for a decade then 1.5–2.0 °C for an additional 4 years before sampling. It is possible that 4 years were not long enough to invoke the critical temperature threshold for soil warming that could induce structural changes in taxonomy. Fourth, microbial composition is substantially impacted by plant and soil geochemical variables, such as plant standing biomass, soil organic carbon, pH, soil moisture, and plant coverage^50–52^, but these variables did not significantly change with warming in our study. Therefore, our results showed that a 1–2 °C elevation in soil temperature, even applied over 14 years, did not have a detectable effect on the overall composition of the soil microbial community.

Although fungal taxonomy was not analyzed in this study, we used GeoChip 5.0 to examine functional gene composition derived from bacteria, fungi, and archaea (Supplementary Fig. 3 and Supplementary Table 2). Bacteria usually comprise the majority of microbial biomass in grassland soils^53^, but there is accumulating evidence highlighting the importance of fungal functional roles for grassland ecosystem processes^38,54^. Our results showed that the compositions of archaeal, bacterial, and fungal functional genes were significantly affected by warming. Similarly, in a grassland study of Inner Mongolia, China, functional gene communities of bacteria, archaea, and fungi were changed by light-intensity livestock grazing while bacterial community composition remained unchanged^55^. Bacterial, archaeal, or fungal organisms typically encompass thousands of functional genes, showing a high dimensionality of the functional profile. Most functional genes are not as conserved as the taxonomic biomarker genes (e.g., 16S rRNA gene for bacteria and archaea, and 18S rRNA gene for fungi). Therefore, functional genes are influenced by environmental conditions, leading to high sensitivity to environmental changes.

However, no significant changes were detected in the composition of C cycling genes after correcting error rates for multiple comparisons using Benjamini–Hochberg correction (Table 2), which could be controversial since the number of tests for error rate correction was arbitrary and variable^56–58^ and prone to higher type II errors^56,59^. Increased gene abundances related to the degradation of labile C but decreased gene abundances related to the degradation of recalcitrant C by long-term warming could affect soil C storage^39,60,61^, though soil mineral control also plays a key role in the retention of soil C. Here, warming significantly altered functional gene composition (Supplementary Fig. 1b and Supplementary Table 2), consistent with results from other long-term warming experiments conducted in a tall-grass prairie in the Great Plains of Central Oklahoma, USA, which detected strong microbial functional responses to warming^62,63^. We found that genes related to labile C degradation pathways increased in warmed plots, whereas those related to recalcitrant C degradation decreased (Fig. 1). This is consistent with results found for other ecosystems, which showed that microbial potential for the metabolization of labile soil C increased in response to warming while that for recalcitrant soil C decreased^62,64^. In addition, a simulated warming study in the alpine grassland of Tibetan plateau showed that relative abundances of catabolic genes associated with more recalcitrant C substrates decreased after warming^60^. A recent study found that 2-year warming increased the total abundance and functional capacities of all potential recalcitrant decomposers in the Alaska tundra^65^. Therefore, the responses of ecosystems to climate warming are complicated, which vary across ecosystem types and the duration of field experiments. Changes in C functional genes might be translated into ecosystems functions, as DNA-based abundances of those genes have been used for assessing CO_2_ efflux^66,67^.

Warming increased the relative abundance of *nifH* gene (Fig. 2), suggesting that warming might enhance microbial N_2_-fixation capacity. This result corroborates those from two long-term warming studies conducted in a tall-grass prairie ecosystem in the Great Plains of Central Oklahoma^63^ and a tundra region in Alaska, USA^68^, implying higher N_2_-fixation capacity in response to soil warming. In contrast, warming decreased the relative abundances of many other functional genes related to N cycling. Among those, warming decreased three denitrification genes (*narG*, *nirS*, and *nosZ*), which translated to a 17.7% lower, albeit insignificant, potential for denitrification in our study (Supplementary Table 3). However, potential nitrification rates remained unchanged (Supplementary Table 3). Quantitative linkages between functional gene abundances and process rates have been reported for *amoA* gene encoding ammonia monooxygenase subunit A^69–71^, which mediates the first and rate-limiting step of nitrification in most grasslands. Accordingly, no changes in *amoA* genes were observed here. In contrast, there was a significant decrease in the relative abundance of *hao* gene encoding hydroxylamine oxygenase, which mediates the subsequent step of oxidizing ammonia to nitrite through the intermediate hydroxylamine (Fig. 2). There are possible reasons for inconsistency between gene abundances and process measurements. First, we only detected 46 *hao* genes, far fewer than the 573 *amoA* genes we report, which suggests a higher degree of functional redundancy for *amoA* and the process catalyzed by this gene than that for *hao*. Second, potential nitrification rates may not be indicative of in situ nitrification rates since the environmental conditions are different from those in the field. Third, a previous study conducted at the same site has shown that warming effects on the potential for ammonium oxidation, nitrite oxidation, and denitrification are only detectable when warming has caused changes larger than 30%^33^. In addition, it was shown that long-term warming could increase rates of N cycling^8,62,63,71^. In this study, we found the reverse situation (i.e., that the relative abundances of most of the key genes involved in N cycling decreased; Fig. 2). This calls for further study, because decreased potentials for key functions involved in N cycling may have important consequences on N cycling in the long term.

Our results indicate that while microbial functional gene abundances and composition are remarkably sensitive to a subtle disturbance of moderate warming, microbial taxonomic composition is remarkably resilient (Supplementary Fig. 1 and Supplementary Table 2). One possible explanation is that functional interchange across different taxa (horizontal gene transfer) and adaptive loss or gain of function through evolution (vertical gene transfer) can collectively result in the conspicuous decoupling of microbial functions from phylogenetic position^72,73^. In addition, we calculated the effective size of warming treatment as the percentages of relative abundance changes: (*X*_t_−*X*_c_)/*X*_c_ × 100%, where *X*_t_ is the mean relative abundance of gene/mean relative abundance of OTU in the treatment, and *X*_c_ is the mean relative abundance of gene/mean relative abundance of OTU in the control. The smallest detectable effective size for functional gene abundances was 2.5%, meaning that all changes <2.5% were insignificant. In contrast, no significant change was observed for any OTUs, which could be attributed to the finding that 16S rRNA gene amplicon sequencing has lower sensitivity than GeoChip^55,64^. However, many questions remain unaddressed. How do environmental conditions differentially interact with taxonomic composition and functional genes? What drives the taxonomic variation within individual functional groups? Can the concept of functional redundancy be used to explain the decoupling of microbial taxonomy and functional genes? In addition, technologies may also play a role. If one performs a metagenomic analysis and extracts taxonomic and functional gene information from the same dataset, will stronger association between microbial taxonomy and functional genes be observed? Future endeavors are needed to tackle those questions.

In summary, here we examine long-term warming effects on soil microbial communities in a Mediterranean-type grassland. We found that warming-induced significant changes in functional gene abundances but had no detectable effect on taxonomic composition of the soil microbial community, suggesting that a functional gene-centric approach to measure and explain responses of microbial communities to environmental changes was more sensitive. Several functional genes involved in the degradation of labile C increased and recalcitrant C decreased by warming, whereas many functional genes involved in N cycling decreased in response to warming. This highlights the importance of taking account of microbial functional potentials in examining ecological consequences of long-term warming. However, an important caveat to bear in mind is that this study lacks much of the insight into interactions that exist between community composition, functional gene expression or activity, and soil processes since there were no measures of soil processes or assessment of gene expression. Future studies should include metatranscriptomic, metaproteomic, or enzymatic activity approaches to generate a better understanding about the warming effects on soil microbial communities.

## Methods

### Site description and experimental design

We conducted this study in an annual grassland ecosystem at the JRGCE site in the San Francisco Bay area, which is located in the eastern foothills of the Santa Cruz Mountains (37°40′ N, 122°22′ W, elevation 150 m), CA, USA. In this Mediterranean-climate ecosystem, plants germinate in October or November and senesce during May and June. The plant community is typical of a Californian annual grassland, and is dominated by annual grasses *Avena barbata*, *Avena fatua*, *Bromus hordeaceus*, and *Lolium multiflorum*^36,37^, and annual forbs *Geranium dissectum*, *Erodium botrys*, and *Crepis vesicaria*.

This warming experiment is a part of a multi-factor global climate change experiment initiated in 1998, which also involves manipulation of atmospheric CO_2_, N deposition, and precipitation^74^, and an additional fire treatment. The experiment comprised 32 circular grassland plots (2-m diameter plots), each being divided into four quadrants of 0.78 m^2^, resulting in a total of 128 subplots. Every quadrant was separated by a fiberglass barrier (0.5 m belowground) to discourage roots and resources from escaping the quadrants and by a mesh (0.5 m aboveground) to prevent plants, seeds, and litter from crossing quadrant boundaries. Only control subplots (*n* = 4) and continuously warming subplots (*n* = 4) were used in the present study; in those subplots, all other global change factors were at ambient levels. Warming was achieved with an array of four overhead infrared heaters (165 × 15 cm; Kalglo Electronics, Bethlehem, PA), which were suspended 1.5 m above the ground in each warmed plot (each heater centered over one quadrant). Temperature was increased by 100 W heaters, resulting in warming of 0.8–1.0 °C at the soil surface from 1998 to the end of the 2008–2009 growing season. Then temperature was increased by 250 W heaters, resulting in a warming of 1.5–2.0 °C at the soil surface since the beginning of the 2009–2010 growing season. The warming level was within the range of estimates for the second half of this century^75^. Since the spectrum of red: far-red is 622–800 nm, infrared heaters may not have effects on red: far-red received by canopy. Dummy heater hoods were also implemented in control plots to reproduce any shading effects without warming. Soil temperature was measured hourly in each quadrant using thermocouples buried at the depth of 10 cm^33^, and hourly soil temperatures were averaged over the entire month or over the entire year (annual mean).

Soil samples were collected in control and warmed plots on April 26–27, 2012 (i.e., 14 years after the warming treatment started), corresponding to the peak period of plant growth in the four control subplots and in the four warming subplots. One soil core (5 cm in width and 7 cm in depth) was taken from each quadrant. After removing visible plant roots and stones, soil samples were thoroughly mixed and sieved through a 2 mm mesh. Then soil samples were divided into three parts: one part was stored for a few days at 4 °C before conducting nitrifying and denitrifying enzyme activity assays, a second part was stored at −20 °C prior to a range of soil geochemical measurements, and a third part was stored at −80 °C prior to DNA extraction.

### Measurements of plant variables

As described previously^29,36^, aboveground plant material was collected on May 5, 2012 from a 141 cm^2^ area of each quadrant. Total aboveground biomass (TAGB) was a sum of the biomass of individual plant species. The biomass of individual plant species was combined into functional groups, including annual grass biomass (AGB), annual forbs biomass (AFB), perennial forbs biomass (PFB), and litter biomass (LB). Fine root biomass (FRB) was determined by separating roots out of soil cores taken in the same area of the aboveground biomass harvest. Total shallow root biomass (TSRB) was measured by collecting roots at the depth of 0–15 cm in soil cores. All biomass was oven-dried (70 °C) for 72 h before weighing^29,76^.

### Measurements of soil geochemical variables and soil microbial activities

Soil moisture of each sample was determined by drying 10 g of fresh soil for a week (or until no change in mass) at 105 °C. Soil pH was measured by suspending 5 g soil in 10 ml distilled water and measuring with the glass combination electrode of an Accumet pH meter (Thermo Fisher Scientific Inc., Waltham, Massachusetts, USA). Total soil C (TC) and total soil N (TN) were measured by dry combustion analysis^77^ with a Carlo Erba Strumentazione Model 1500 Series I analyzer (Carlo Erba Strumentazione, Rodano, Italy). Soil C/N ratio was calculated as the ratio of TC to TN. Soil ammonium (NH_4_^+^) and nitrate (NO_3_^‒^) concentrations were determined by preparing 5 g of fresh soil, shaken in 50 ml of 2 M KCl solution. Then extracts were measured using a SEAL Automated Segmented Flow analyzer (SEAL Analytical Inc, Mequon, Wisconsin, USA) at Loyola University, Chicago, IL, USA.

Nitrifying enzyme activity reflects the pool of nitrifying enzymes, which function to oxidize NH_4_^+^ into nitrite (NO_2_^−^) and nitrate (NO_3_^−^) under optimal conditions. We incubated sterile Erlenmeyer flasks containing 45 ml of 1 mM phosphate buffer (pH 7.4), 0.04 ml of 0.25 M (NH_4_)_2_SO_4_, and fresh soil sub-samples (5 g of equivalent dry soil) at 25 °C on a shaker with constant agitation at 150 rpm^78^. The 5 ml solution was sampled every 2 h for 10 h and stored at −20 °C until analysis of nitrite and nitrate concentrations in a DIONEX ion chromatograph system (Sunnyvale, CA, USA) equipped with an AS11-HC analytical column^79^. Potential nitrification rates were determined from the slope of the linear regression of nitrite plus nitrate concentration on time.

Denitrifying enzyme activity was measured as the nitrous oxide (N_2_O) production rate by soil samples amended with NO_3_^−^ and labile C^80^. Fresh soil (5 g of equivalent dry soil) was placed in a 150 ml plasma flask containing 5 mg glucose, 5 mg glutamic acid, and 0.8 mg KNO_3_. We inhibited N_2_O reductase with a 90:10 N_2_:acetylene mixture. N_2_O concentrations were analyzed every 2 h for 8 h by an Agilent P200 Micro gas chromatograph (Agilent Technologies, Palo Alto, CA, USA) equipped with an electron capture detector^33^. Potential denitrification rates were determined from the slope of the linear regression of N_2_O concentration on time.

### DNA extraction, purification, and quantification

DNA was extracted from 5 g frozen soil by a freeze-grinding mechanical lysis method as previously described^81^, and then purified using gel electrophoresis with a 0.5% low-melting-point agarose. DNA bands were visualized under UV light and were excised from the gel using a sterile cutter as quickly as possible. Then DNA was eluted in 15 ml double distilled water followed by phenol–chloroform–butanol extraction. DNA was dissolved in sterile, nuclease-free water and stored at −20 °C for only a few days before PCR amplification. We assessed DNA purity using the absorbance ratios of A_260_/A_280_ (>1.8) and A_260_/A_230_ (>1.7). The final DNA concentration was measured with a PicoGreen® method (BMG Labtech, Jena, Germany).

### MiSeq sequencing and preprocessing

We used the F515 (5′-GTGCCAGCMGCCGCGG-3′) and R806 (3′-TAATCTWTGGGVHCATCAG-5′) primers to amplify the V4 hypervariable region of bacterial and archaeal 16S rRNA gene. Three replicates of PCR reactions were performed for each sample, which were then pooled together before sequencing on MiSeq platform (Illumina, San Diego, CA) as previously described^82^. Sequencing data were processed and analyzed with the Microbial Ecology Community Pipeline (http://zhoulab5.rccc.ou.edu:8080). Paired raw sequences were identified by unique 12-mer barcodes and merged into longer reads using FLASH^83^. Unqualified sequences were trimmed by Btrim based on quality scores (>20). Sequences were further trimmed to the length of 200–300 bp, and chimera sequences were removed using UCHIME (version 5.2.32)^84^. Reads were assigned to taxonomic units (OTUs) using UPARSE (version usearch 7.0.1001) filtered at 97% nucleotide identity, and a representative sequence of each OTU was generated. To correct for differences in sequencing depth, 28,562 sequences were randomly resampled for each sample before performing the downstream analyses. Sequences observed only once (singletons) were removed to improve data reliability.

### Hybridization with GeoChip 5.0 and raw data processing

A total of 1 μg DNA was labeled with Cy-3 dye by random priming and hybridized with GeoChip 5.0. The microarray hybridization, scanning, and image processing were conducted as previously described^60,85^. Raw GeoChip data processing was performed with the following steps: (i) remove poor-quality spots with a signal-to-noise ratio (SNR) <2.0, (ii) remove genes detected only in one replicate out of four samples from the same treatment, (iii) perform logarithmic transformation of the signal intensity of each gene, and (iv) normalize the data by dividing the transformed signal intensities of each gene by the mean intensity in the sample.

### Statistical analyses

Statistical analyses were performed with R software (v.3.1.0.; R foundation for statistical computing). Functional gene and taxonomic community composition differences were examined by non-metric multidimensional scaling (NMDS) based on the Bray–Curtis distances. Nonparametric multivariate statistical tests (ANOSIM, Adonis, and MRPP) based on different algorithms were performed to examine statistical significance. Specifically, Adonis is an analysis-of-variance analog for multivariate data, which returns an R^2^ with a Monte Carlo-permuted *P* value, ANOSIM is based on rank-order distances and effectively uncovers apparent differences in clusters, while MRPP uses original resemblances and thus is more sensitive to subtle differences across experimental groups. Although they often yield similar results, there are occasions that their results differ. Therefore, it is a popular practice to conduct all three tests simultaneously to show the reliability of the results.

The significance of differences in relative abundances of genes between warmed and control plots were analyzed by two-tailed paired Student’s *t*-tests. Normality of the data was verified using Shapiroe–Wilk’s tests. Almost all of the genes showed normal distribution and low skewnesses. Only a gene encoding inulinase violates the assumptions of normal distribution, thus the nonparametric analysis, Mann–Whitney U test, was performed. Alpha-diversity indices, including Shannon index (*H*), Simpsons diversity index (*D*), and Pielou’s evenness (*J*), were calculated based on the richness and relative abundance data as follows:

1 $$H = - \mathop {\sum}\limits_{i = 1}^S {p_i\ln p_i} ,$$

2 $$D = {\sum} {\left( {P_i} \right)^2} ,$$

3 $$J = H/\ln \left( S \right),$$

where _*pi*_ = *n*_*i*_/*N*, *n*_*i*_ is the abundance of the *i*th OTU (gene), and *N* is the total abundance of all OTUs (genes) in the sample. *S* is the species (gene) richness. The *P* value was adjusted for false discovery rate of 0.05 using the Benjamini–Hochberg method^86^, which was widely used in environmental genomics studies^64,87^.

### Reporting summary

Further information on research design is available in the Nature Research Reporting Summary linked to this article.

## Supplementary information

Supplementary Information

Reporting Summary

## Data Availability

GeoChip data are available online (www.ncbi.nlm.nih.gov/geo/) with the accession number GSE107168. MiSeq data are available in NCBI SRA database with the accession number SRP126539.

## References

[1] IPCC Climate Change 2013. *The Physical Science Basis: Working Group I Contribution to the Fifth Assessment Report of the Intergovernmental Panel on Climate Change* (Cambridge University Press, 2013).

[2] Baldwin AH, Jensen K, Schönfeldt M (2014). Warming increases plant biomass and reduces diversity across continents, latitudes, and species migration scenarios in experimental wetland communities. Glob. Change Biol..

[3] Niu S (2008). Water-mediated responses of ecosystem carbon fluxes to climatic change in a temperate steppe. N. Phytol..

[4] Rustad LE (2008). The response of terrestrial ecosystems to global climate change: towards an integrated approach. Sci. Total Environ..

[5] Natali SM, Schuur EAG, Rubin RL (2012). Increased plant productivity in Alaskan tundra as a result of experimental warming of soil and permafrost. J. Ecol..

[6] Bertrand R (2011). Changes in plant community composition lag behind climate warming in lowland forests. Nature.

[7] Wang S (2012). Effects of warming and grazing on soil N availability, species composition, and ANPP in an alpine meadow. Ecology.

[8] Bai E (2013). A meta-analysis of experimental warming effects on terrestrial nitrogen pools and dynamics. N. Phytol..

[9] Belay-Tedla A, Zhou X, Su B, Wan S, Luo Y (2009). Labile, recalcitrant, and microbial carbon and nitrogen pools of a tallgrass prairie soil in the US Great Plains subjected to experimental warming and clipping. Soil Biol. Biochem..

[10] Melillo JM (2011). Soil warming, carbon–nitrogen interactions, and forest carbon budgets. Proc. Natl Acad. Sci. USA.

[11] Barnard R (2004). Atmospheric CO_2_ elevation has little effect on nitrifying and denitrifying enzyme activity in four European grasslands. Glob. Change Biol..

[12] Griffiths BS (2001). An examination of the biodiversity–ecosystem function relationship in arable soil microbial communities. Soil Biol. Biochem..

[13] Jurburg SD (2017). Autogenic succession and deterministic recovery following disturbance in soil bacterial communities. Sci. Rep..

[14] Waldrop MP, Firestone MK (2006). Response of microbial community composition and function to soil climate change. Microb. Ecol..

[15] Zogg GP (1997). Compositional and functional shifts in microbial communities due to soil warming. Soil Sci. Soc. Am. J..

[16] Yang Y (2014). The microbial gene diversity along an elevation gradient of the Tibetan grassland. ISME J..

[17] Falkowski PG, Fenchel T, Delong EF (2008). The microbial engines that drive earth’s biogeochemical cycles. Science.

[18] Luo Y, Wan S, Hui D, Wallace LL (2001). Acclimatization of soil respiration to warming in a tall grass prairie. Nature.

[19] Melillo J (2002). Soil warming and carbon-cycle feedbacks to the climate system. Science.

[20] Rustad L (2001). A meta-analysis of the response of soil respiration, net nitrogen mineralization, and aboveground plant growth to experimental ecosystem warming. Oecologia.

[21] Chen J (2015). Stronger warming effects on microbial abundances in colder regions. Sci. Rep..

[22] Treseder KK (2012). Integrating microbial ecology into ecosystem models: challenges and priorities. Biogeochemistry.

[23] Wieder WR, Bonan GB, Allison SD (2013). Global soil carbon projections are improved by modelling microbial processes. Nat. Clim. Change.

[24] Giorgi F (2006). Climate change hot-spots. Geophys. Res. Lett..

[25] Giorgi F, Lionello P (2008). Climate change projections for the Mediterranean region. Glob. Planet Change.

[26] Cayan DR, Maurer EP, Dettinger MD, Tyree M, Hayhoe K (2008). Climate change scenarios for the California region. Clim. Change.

[27] Cleland EE, Chiariello NR, Loarie SR, Mooney HA, Field CB (2006). Diverse responses of phenology to global changes in a grassland ecosystem. Proc. Natl Acad. Sci. USA.

[28] Zavaleta ES (2003). Grassland responses to three years of elevated temperature, CO_2_, precipitation, and N deposition. Ecol. Monogr..

[29] Dukes JS (2005). Responses of grassland production to single and multiple global environmental changes. PLoS Biol..

[30] Henry HAL, Juarez JD, Field CB, Vitousek PM (2005). Interactive effects of elevated CO_2_, N deposition and climate change on extracellular enzyme activity and soil density fractionation in a California annual grassland. Glob. Change Biol..

[31] Horz HP, Barbrook A, Field CB, Bohannan BJ (2004). Ammonia-oxidizing bacteria respond to multifactorial global change. Proc. Natl Acad. Sci. USA.

[32] Horz HP, Rich V, Avrahami S, Bohannan BJM (2005). Methane-oxidizing bacteria in a California upland grassland soil: diversity and response to simulated global change. Appl. Environ. Microbiol..

[33] Niboyet A (2011). Testing interactive effects of global environmental changes on soil nitrogen cycling. Ecosphere.

[34] Barnard R (2006). Several components of global change alter nitrifying and denitrifying activities in an annual grassland. Funct. Ecol..

[35] Rillig MC, Wright SF, Shaw MR, Field CB (2002). Artificial climate warming positively affects arbuscular mycorrhizae but decreases soil aggregate water stability in an annual grassland. Oikos.

[36] Gutknecht JL, Field CB, Balser TC (2012). Microbial communities and their responses to simulated global change fluctuate greatly over multiple years. Glob. Change Biol..

[37] Liang C, Balser TC (2012). Warming and nitrogen deposition lessen microbial residue contribution to soil carbon pool. Nat. Commun..

[38] DeAngelis KM (2015). Long-term forest soil warming alters microbial communities in temperate forest soils. Front. Microbiol..

[39] Melillo JM (2017). Long-term pattern and magnitude of soil carbon feedback to the climate system in a warming world. Science.

[40] Luo Y (2011). Coordinated approaches to quantify long-term ecosystem dynamics in response to global change. Glob. Change Biol..

[41] Louca S, Parfrey LW, Doebeli M (2016). Decoupling function and taxonomy in the global ocean microbiome. Science.

[42] Louca S (2018). Function and functional redundancy in microbial systems. Nat. Ecol. Evol..

[43] Cruz-Martínez K (2009). Despite strong seasonal responses, soil microbial consortia are more resilient to long-term changes in rainfall than overlying grassland. ISME J..

[44] Parker SS, Schimel JP (2011). Soil nitrogen availability and transformations differ between the summer and the growing season in a California grassland. Appl. Soil Ecol..

[45] Cabrol L (2016). Management of microbial communities through transient disturbances enhances the functional resilience of nitrifying gas-biofilters to future disturbances. Environ. Sci. Technol..

[46] Guo X (2018). Climate warming leads to divergent succession of grassland microbial communities. Nat. Clim. Change.

[47] Strong AL, Johnson TP, Chiariello NR, Field CB (2017). Experimental fire increases soil carbon dioxide efflux in a grassland long-term multifactor global change experiment. Glob. Change Biol..

[48] Zavaleta ES (2003). Plants reverse warming effect on ecosystem water balance. Proc. Natl Acad. Sci. USA.

[49] Abbasi AO (2020). Reviews and syntheses: soil responses to manipulated precipitation changes–an assessment of meta-analyses. Biogeosciences.

[50] Fierer N (2012). Cross-biome metagenomic analyses of soil microbial communities and their functional attributes. Proc. Natl Acad. Sci. USA.

[51] Drenovsky R, Vo D, Graham K, Scow K (2004). Soil water content and organic carbon availability are major determinants of soil microbial community composition. Microb. Ecol..

[52] He Z (2011). The phylogenetic composition and structure of soil microbial communities shifts in response to elevated carbon dioxide. ISME J..

[53] De Vries FT, Hoffland E, van Eekeren N, Brussaard L, Bloem J (2006). Fungal/bacterial ratios in 663 grasslands with contrasting nitrogen management. Soil Biol. Biochem..

[54] Antoninka A, Reich PB, Johnson NC (2011). Seven years of carbon dioxide enrichment, nitrogen fertilization and plant diversity influence arbuscular mycorrhizal fungi in a grassland ecosystem. N. Phytol..

[55] Ma X (2019). Microbial functional traits are sensitive indicators of mild disturbance by lamb grazing. ISME J..

[56] Feise RJ (2002). Do multiple outcome measures require p-value adjustment?. BMC Med. Res. Methodol..

[57] Savitz DA, Olshan AF (1995). Multiple comparisons and related issues in the interpretation of epidemiologic data. Am. J. Epidemiol..

[58] Perneger TV (1998). What’s wrong with Bonferroni adjustments. BMJ.

[59] Rothman KJ (1990). No adjustments are needed for multiple comparisons. Epidemiology.

[60] Yue H (2015). The microbe-mediated mechanisms affecting topsoil carbon stock in Tibetan grasslands. ISME J..

[61] He Z (2010). Metagenomic analysis reveals a marked divergence in the structure of belowground microbial communities at elevated CO_2_. Ecol. Lett..

[62] Luo C (2014). Soil microbial community responses to a decade of warming as revealed by comparative metagenomics. Appl. Environ. Microbiol..

[63] Zhou J (2011). Microbial mediation of carbon-cycle feedbacks to climate warming. Nat. Clim. Change.

[64] Xue K (2016). Tundra soil carbon is vulnerable to rapid microbial decomposition under climate warming. Nat. Clim. Change.

[65] Tao X (2020). Winter warming in Alaska accelerates lignin decomposition contributed by Proteobacteria. Microbiome.

[66] Trivedi P (2016). Microbial regulation of the soil carbon cycle: evidence from gene–enzyme relationships. ISME J..

[67] Zhao M (2014). Microbial mediation of biogeochemical cycles revealed by simulation of global changes with soil transplant and cropping. ISME J..

[68] Feng J (2019). Long-term warming in Alaska enlarges the diazotrophic community in deep soils. mBio.

[69] Liu S (2015). The interactive effects of soil transplant into colder regions and cropping on soil microbiology and biogeochemistry. Environ. Microbiol..

[70] Zhao M (2016). Zonal soil type determines soil microbial responses to maize cropping and fertilization. mSystems.

[71] Luo Y (2007). Terrestrial carbon-cycle feedback to climate warming. Annu. Rev. Ecol. Evol. Syst..

[72] Green JL, Bohannan BJM, Whitaker RJ (2008). Microbial biogeography: from taxonomy to traits. Science.

[73] Martiny, J. B. H., Jones, S. E., Lennon, J. T. & Martiny, A. C. Microbiomes in light of traits: a phylogenetic perspective. *Science* **350**, aac9323 (2015). 10.1126/science.aac9323 26542581

[74] Shaw MR (2002). Grassland responses to global environmental changes suppressed by elevated CO_2_. Science.

[75] Hayhoe K (2004). Emissions pathways, climate change, and impacts on California. Proc. Natl Acad. Sci. USA.

[76] Zavaleta ES, Shaw MR, Chiariello NR, Mooney HA, Field CB (2003). Additive effects of simulated climate changes, elevated CO_2_, and nitrogen deposition on grassland diversity. Proc. Natl Acad. Sci. USA.

[77] Nelson, D. W. et al. in *Methods of Soil Analysis. Part 3: Chemical Methods*, Vol. 9 (Soil Science Society of America, Inc., American Society of Agronomy, Inc., 1996).

[78] Lensi R, Mazurier S, GourbiÉre F, Josserand A (1986). Rapid determination of the nitrification potential of an acid forest soil and assessment of its variability. Soil Biol. Biochem..

[79] Avrahami S, Bohannan BJM (2009). N_2_O emission rates in a California meadow soil are influenced by fertilizer level, soil moisture and the community structure of ammonia-oxidizing bacteria. Glob. Change Biol..

[80] Smith MS, Tiedje JM (1979). Phases of denitrification following oxygen depletion in soil. Soil Biol. Biochem..

[81] Zhou J, Bruns MA, Tiedje JM (1996). DNA recovery from soils of diverse composition. Appl. Environ. Microbiol..

[82] Caporaso JG (2012). Ultra-high-throughput microbial community analysis on the Illumina HiSeq and MiSeq platforms. ISME J..

[83] Magoč T, Salzberg SL (2011). FLASH: fast length adjustment of short reads to improve genome assemblies. Bioinformatics.

[84] Edgar RC, Haas BJ, Clemente JC, Quince C, Knight R (2011). UCHIME improves sensitivity and speed of chimera detection. Bioinformatics.

[85] Wu L (2017). Alpine soil carbon is vulnerable to rapid microbial decomposition under climate cooling. ISME J..

[86] Benjamini Y, Hochberg Y (1995). Controlling the false discovery rate: a practical and powerful approach to multiple testing. J. R. Stat. Soc. Ser. B Methodol..

[87] Pepe-Ranney C (2015). Non-cyanobacterial diazotrophs mediate dinitrogen fixation in biological soil crusts during early crust formation. ISME J..

