# Supplementary material for: Long-term warming in a Mediterranean-type grassland affects soil bacterial functional potential but not bacterial taxonomic composition
Source: NPJ Biofilms Microbiomes. 2021 Feb 8;7:17. doi: 10.1038/s41522-021-00187-7 (PMC7870951; doi:10.1038/s41522-021-00187-7)
Supplement: Supplementary file 1 — Supplementary Information [file 41522_2021_187_MOESM1_ESM.pdf]

SUPPLEMENTARY FILE

Long-term warming in a Mediterranean-type grassland affects soil bacterial functional potential but not bacterial taxonomic composition

Ying Gao[1,2], Junjun Ding[2,3], Mengting Yuan[4], Nona Chiariello[5], Kathryn Docherty[6], Chris Field[5], Qun Gao[2], Baohua Gu[7], Jessica Gutknecht[8,9], Bruce A. Hungate[10,11], Xavier Le Roux[12], Audrey Niboyet[13,14], Qi Qi[2], Zhou Shi[4], Jizhong Zhou[2,4,15] and Yunfeng Yang[2*]

[1]Institute of Desertification Studies, Chinese Academy of Forestry, Beijing, 100091, China
[2]State Key Joint Laboratory of Environment Simulation and Pollution Control, School of Environment, Tsinghua University, Beijing 100084, China
[3]Key Laboratory of Dryland Agriculture, Ministry of Agriculture of the People's Republic of China, Institute of Environment and Sustainable Development in Agriculture, Chinese Academy of Agricultural Sciences, Beijing 100081, China
[4]Institute for Environmental Genomics and Department of Microbiology and Plant Biology, University of Oklahoma, Norman, OK 73019, USA
[5]Department of Global Ecology, Carnegie Institution for Science, Stanford, CA 94305, USA
[6]Department of Biological Sciences, Western Michigan University, Kalamazoo, MI 49008, USA
[7]Environmental Sciences Division, Oak Ridge National Laboratory, Oak Ridge, TN 37831, USA
[8]Department of Soil Ecology, Helmholtz Centre for Environmental Research - UFZ, Halle 06120, Germany
[9]Department of Soil, Water, and Climate, University of Minnesota, Twin Cities, Saint Paul, MN 55104, USA
[10]Center for Ecosystem Science and Society, Northern Arizona University, Flagstaff, AZ 86011, USA
[11]Department of Biological Sciences, Northern Arizona University, Flagstaff, AZ 86011, USA
[12]Mirobial Ecology Centre LEM, INRA, CNRS, University of Lyon, University Lyon 1, UMR INRA 1418, 43 boulevard du 11 novembre 1918, 69622 Villeurbanne, France
[13]Institut d'Ecologie et des Sciences de l'Environnement de Paris (Sorbonne Université, CNRS, INRA, IRD, Université Paris Diderot, UPEC), 4 place Jussieu, 75005, Paris, France
[14]AgroParisTech, 75005, Paris, France
[15]Earth Sciences Division, Lawrence Berkeley National Laboratory, Berkeley, CA 94720, USA
*To whom correspondence may be addressed. E-mail: yangyf@tsinghua.edu.cn;

Supplementary figure

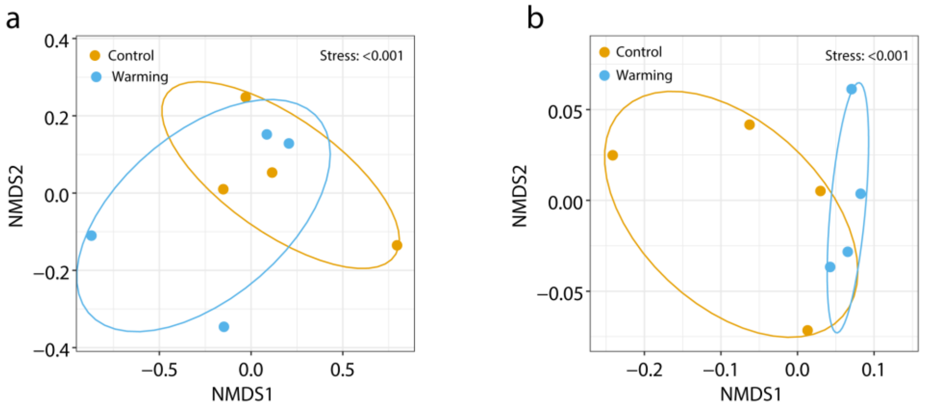

Supplementary Figure 1. Community composition displayed using non-metric multidimensional scaling (NMDS) of (a) taxonomic composition and (b) functional genes of the microbial community in control and warming samples.

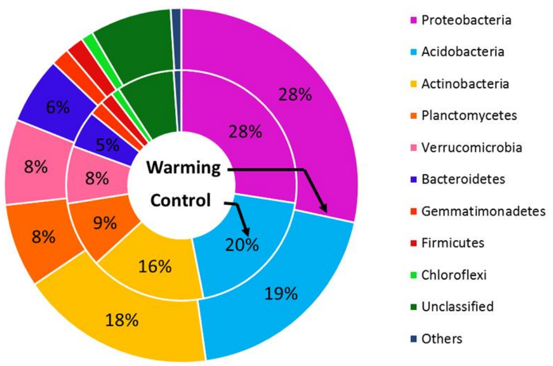

Supplementary Figure 2. Relative abundances of the different phyla in the control and warmed plots. Rings represent the average abundances of each phylum that account for at least 1% of total abundances.

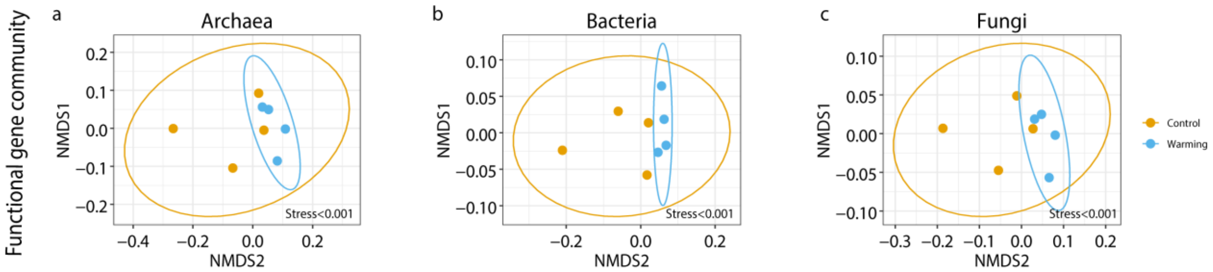

Supplementary Figure 3. Community composition displayed using non-metric multidimensional scaling (NMDS) of (a) archaeal functional genes, (b) bacterial functional gene composition, and (c) fungal functional gene composition in control and warmed plots.

Supplementary Table 1. Summary of operational taxonomic units (OTUs) and functional gene diversities[a].

	16S rRNA gene sequencing		GeoChip	
	**Control**	**Warming**	**Control**	**Warming**
**Shannon index**	8.175±0.008	8.301±0.116	10.85±0.03	10.87±0.01
**Simpson index**	1354.74±63.59	1371.59±134.44	51225.5±1477.13	52673.38±603.37
**Pielou evenness**	0.907±0.001	0.908±0.004	1±0	1±0

[a] There are no significant changes between warmed and control plots.

Supplementary Table 2. Significance tests of the effects of warming on the overall taxonomic and functional gene composition with three nonparametric statistical methods

**Statistical approaches**		**Adonis**[a]	**ANOSIM**[b]	**MRPP**[c]
**Whole taxonomic community**	**Statistic**	1.044	-0.104	0.484
	***P* -value**[d]	0.323	0.851	0.403
**Whole functional gene community**	**Statistic**	2.69	0.365	0.106
	***P* -value**	**0.001****	**0.030****	**0.034****
**Bacterial functional gene community**	**Statistic**	0.282	0.292	0.08
	***P* -value**	**0.034****	**0.03****	**0.024****
**Archaeal functional gene community**	**Statistic**	0.284	0.313	0.081
	***P* -value**	**0.026****	**0.025****	**0.03****
**Fungal functional gene community**	**Statistic**	0.302	0.323	0.092
	***P* -value**	**0.031****	**0.025****	**0.031****

[a]Non-parametric multivariate analysis of variance (MANOVA) with the adonis function.
[b]Analysis of similarities.
[c]Multiple response permutation procedure, a nonparametric procedure that does not depend on assumptions such as normally distributed data or homogeneous variances, but rather depends on the internal variability of the data.
[d]Significant effects are indicated in bold font. ******, $P<0.05$.

Supplementary Table 3. The mean values of nitrifying enzyme activity (NEA) and denitrifying enzyme activity (DEA). No significant changes are observed between warmed and control plots by two-tailed paired Student's *t*-tests.

	**Control**	**Warming**
NEA ($\times 10^{-4}$mg^{-1} N g^{-1}h^{-1})	1.121±0.211	1.200±0.170
DEA ($\times 10^{-4}$mg^{-1} N g^{-1}h^{-1})	6.625±0.990	5.454±1.034
