# Supplementary material for: Long-term warming in a Mediterranean-type grassland affects soil bacterial functional potential but not bacterial taxonomic composition
Source: NPJ Biofilms Microbiomes. 2021 Feb 8;7:17. doi: 10.1038/s41522-021-00187-7 (PMC7870951; doi:10.1038/s41522-021-00187-7)
Supplement: Supplementary file 2 — Reporting Summary [file 41522_2021_187_MOESM2_ESM.pdf]

nature research

Corresponding author(s): Yunfeng Yang
Last updated by author(s): Dec 23, 2020

Reporting Summary

Nature Research wishes to improve the reproducibility of the work that we publish. This form provides structure for consistency and transparency in reporting. For further information on Nature Research policies, see our Editorial Policies and the Editorial Policy Checklist.

Statistics

For all statistical analyses, confirm that the following items are present in the figure legend, table legend, main text, or Methods section.

n/a	Confirmed	
☐	☒	The exact sample size (*n*) for each experimental group/condition, given as a discrete number and unit of measurement
☐	☒	A statement on whether measurements were taken from distinct samples or whether the same sample was measured repeatedly
☐	☒	The statistical test(s) used AND whether they are one- or two-sided<br>*Only common tests should be described solely by name; describe more complex techniques in the Methods section.*
☒	☐	A description of all covariates tested
☐	☒	A description of any assumptions or corrections, such as tests of normality and adjustment for multiple comparisons
☐	☒	A full description of the statistical parameters including central tendency (e.g. means) or other basic estimates (e.g. regression coefficient) AND variation (e.g. standard deviation) or associated estimates of uncertainty (e.g. confidence intervals)
☐	☒	For null hypothesis testing, the test statistic (e.g. *F*, *t*, *r*) with confidence intervals, effect sizes, degrees of freedom and *P* value noted<br>*Give P values as exact values whenever suitable.*
☒	☐	For Bayesian analysis, information on the choice of priors and Markov chain Monte Carlo settings
☒	☐	For hierarchical and complex designs, identification of the appropriate level for tests and full reporting of outcomes
☐	☒	Estimates of effect sizes (e.g. Cohen's *d*, Pearson's *r*), indicating how they were calculated

Our web collection on statistics for biologists contains articles on many of the points above.

Software and code

Policy information about availability of computer code

Data collection	MiSeq platform (Illumina Inc., San Diego, CA, USA) and GeoChip 5.0 (Agilent Technologies Inc., Santa Clara, CA, USA) were used to acquire sequence and gene data, respectively
Data analysis	Sequencing data were processed and analyzed with the Microbial Ecology Community Pipeline (http://zhoulab5.rccc.ou.edu:8080). Statistical analyses were performed with R software (v.3.1.0.; R foundation for statistical computing).

For manuscripts utilizing custom algorithms or software that are central to the research but not yet described in published literature, software must be made available to editors and reviewers. We strongly encourage code deposition in a community repository (e.g. GitHub). See the Nature Research guidelines for submitting code & software for further information.

Data

Policy information about availability of data

All manuscripts must include a data availability statement. This statement should provide the following information, where applicable:

- Accession codes, unique identifiers, or web links for publicly available datasets
- A list of figures that have associated raw data
- A description of any restrictions on data availability

GeoChip data are available online (www.ncbi.nlm.nih.gov/geo/) with the accession number GSE107168. MiSeq data are available in NCBI SRA database with the accession number SRP126539.

Field-specific reporting

Please select the one below that is the best fit for your research. If you are not sure, read the appropriate sections before making your selection.

☐ Life sciences ☐ Behavioural & social sciences ☒ Ecological, evolutionary & environmental sciences

For a reference copy of the document with all sections, see nature.com/documents/nr-reporting-summary-flat.pdf

Ecological, evolutionary & environmental sciences study design

All studies must disclose on these points even when the disclosure is negative.

Study description	In this study, we carried out an experiment to investigate taxonomic and functional gene compositions of the soil microbial community after 14 years of warming (0.8-1.0 °C for 10 years and then 1.5-2.0 °C for 4 years) in a Californian grassland.
Research sample	Soil samples were collected from an annual grassland ecosystem at the JRGCE site in the San Francisco Bay area, which is located in the eastern foothills of the Santa Cruz Mountains (37o40' N, 122o22' W, elevation 150 m), CA, USA. This warming experiment is a part of a multi-factor global climate change experiment.
Sampling strategy	We collected 4 replicates for each treatment, which was determined as sufficient sample size.
Data collection	We collected soil samples from control and warmed plots. Nona Chiariello and JRGCE staff recorded the data.
Timing and spatial scale	Soil samples were collected on April 26 – 27th, 2012 (i.e., 14 years after the warming treatment started), corresponding to the peak period of plant growth in the four control subplots and in the four warming subplots.
Data exclusions	No data were excluded from the analyses.
Reproducibility	We collected enough sample replicates to ensure the reproducibility of our results. All attempts to repeat the experiments were successful.
Randomization	Treatments are organized in a randomized block split-plot design.
Blinding	Since we conducted the field investigation, no blinding was relevant for this study.

Did the study involve field work? ☒ Yes ☐ No

Field work, collection and transport

Field conditions	Soil temperature ranged from 15 to 16 degree Celsius and soil moisture is around 7.9%.
Location	The experiment site is located in the eastern foothills of the Santa Cruz Mountains (37o40' N, 122o22' W, elevation 150 m), CA, USA.
Access & import/export	All samples were stored in a portable 4 °C refrigerator. After immediate transportation to the laboratory, each sample was divided into three parts: one part was stored for a few days at 4°C before conducting nitrifying and denitrifying enzyme activity assays, a second part was stored at -20°C prior to a range of soil geochemical measurements, and a third part was stored at -80°C prior to DNA extraction.
Disturbance	We stored the samples in the refrigerator and transported the samples via a car to minimize the disturbance.

Reporting for specific materials, systems and methods

We require information from authors about some types of materials, experimental systems and methods used in many studies. Here, indicate whether each material, system or method listed is relevant to your study. If you are not sure if a list item applies to your research, read the appropriate section before selecting a response.

Materials & experimental systems

n/a	Involved in the study
☒	☐ Antibodies
☒	☐ Eukaryotic cell lines
☒	☐ Palaeontology and archaeology
☒	☐ Animals and other organisms
☒	☐ Human research participants
☒	☐ Clinical data
☒	☐ Dual use research of concern

Methods

n/a	Involved in the study
☒	☐ ChIP-seq
☒	☐ Flow cytometry
☒	☐ MRI-based neuroimaging
